# Response to letter to the editor “Should women have lower thresholds for hypertension diagnosis and treatment”

**DOI:** 10.1038/s41371-023-00839-5

**Published:** 2023-05-12

**Authors:** Fabian Meinert, Costas Thomopoulos, Reinhold Kreutz

**Affiliations:** 1 Charité – Universitätsmedizin Berlin, Institute of Clinical Pharmacology and Toxicology, Berlin, Germany; 2 Department of Cardiology, Helena Venizelou Hospital, Athens, Greece

**Keywords:** Hypertension, Risk factors

## To the Editor:

Brewster et al. have raised an important point that the current hypertension guidelines reviewed by Meinert et al. [1] overlook several sex and gender-specific differences. Women have distinct cardiovascular characteristics, such as a shorter arterial tree, higher heart rate, and earlier reflected arterial pulse waves. These characteristics can result in relatively high central systolic blood pressure and lower diastolic blood pressure, leading to early vascular stiffness, isolated systolic hypertension, lower coronary perfusion, and more microvascular damage. Furthermore, several reproduction-related factors, including uterine fibroids, polycystic ovary syndrome, and endometriosis, are associated with an increased risk of hypertension and other cardiometabolic risk factors in women, as well as a greater risk of major adverse cardiovascular events. Recent studies on women with symptomatic fibroids have revealed abnormalities in the ultrastructure of resistance-sized arteries, even in those considered clinically normotensive. These findings emphasize the importance of addressing sex-specific aspects of hypertension in future guidelines [2].

We appreciate the comments by Brewster et al. on this topic and agree that current hypertension guidelines should address sex and gender-specific differences in more detail including aspects suggested by these authors. Whether or not different thresholds for initiation and targets of blood pressure lowering treatment in women with hypertension could be considered is also an important question. However, the latter depends on the available evidence to do so as supported by dedicated clinical trials. The higher risk of cardiovascular events in normotensive women with uterine fibroids is another interesting point that could be included in risk stratification representing an additional sex-specific difference in the management of individuals with hypertension.
